# The length of uninterrupted CAG repeats in stem regions of repeat disease associated hairpins determines the amount of short CAG oligonucleotides that are toxic to cells through RNA interference

**DOI:** 10.1038/s41419-022-05494-1

**Published:** 2022-12-30

**Authors:** Andrea E. Murmann, Monal Patel, Si-Yeon Jeong, Elizabeth T. Bartom, A. Jennifer Morton, Marcus E. Peter

**Affiliations:** 1 Department of Medicine/Division Hematology/Oncology, Feinberg School of Medicine, Northwestern University, Chicago, IL USA; 2 Department of Biochemistry and Molecular Genetics, Feinberg School of Medicine, Northwestern University, Chicago, IL USA; 3 Department of Preventive Medicine/Division of Biostatistics, Feinberg School of Medicine, Northwestern University, Chicago, IL USA; 4 Department of Physiology, Development and Neuroscience, University of Cambridge, Cambridge, UK; 5 Present Address: Ministry of Food and Drug Safety, Pharmaceutical Safety Bureau, Pharmaceutical Policy Division 187, Cheongju-si, Chungcheongbuk-do Republic of Korea

**Keywords:** Neurodegeneration, Cell death

## Abstract

Extended CAG trinucleotide repeats (TNR) in the genes huntingtin (*HTT*) and androgen receptor (*AR*) are the cause of two progressive neurodegenerative disorders: Huntington’s disease (HD) and Spinal and Bulbar Muscular Atrophy (SBMA), respectively. Anyone who inherits the mutant gene in the complete penetrance range (>39 repeats for HD and 44 for SBMA) will develop the disease. An inverse correlation exists between the length of the CAG repeat and the severity and age of onset of the diseases. Growing evidence suggests that it is the length of uninterrupted CAG repeats in the mRNA rather than the length of poly glutamine (polyQ) in mutant (m)*HTT* protein that determines disease progression. One variant of m*HTT* (loss of inhibition; LOI) causes a 25 year earlier onset of HD when compared to a reference sequence, despite both coding for a protein that contains an identical number of glutamines. Short 21–22 nt CAG repeat (sCAGs)-containing RNAs can cause disease through RNA interference (RNAi). RNA hairpins (HPs) forming at the CAG TNRs are stabilized by adjacent CCG (in HD) or CUG repeats (in SBMA) making them better substrates for Dicer, the enzyme that processes CAG HPs into sCAGs. We now show that cells deficient in Dicer or unable to mediate RNAi are resistant to the toxicity of the *HTT* and *AR* derived HPs. Expression of a small HP that mimics the HD LOI variant is more stable and more toxic than a reference HP. We report that the LOI HP is processed by Dicer, loaded into the RISC more efficiently, and gives rise to a higher quantity of RISC-bound 22 nt sCAGs. Our data support the notion that RNAi contributes to the cell death seen in HD and SBMA and provide an explanation for the dramatically reduced onset of disease in HD patients that carry the LOI variant.

## Introduction

Trinucleotide repeat (TNR) expansions in a number of genes are the cause of many neurodegenerative diseases [1]. The most frequently amplified triplet is CAG (that codes for the amino acid glutamine [Q]), as found in Huntington’s disease (HD) [2], Spinal and Bulbar Muscular Atrophy (SBMA) [3], and many other so-called triplet repeat diseases [4–12]. HD is caused by expansion of a CAG repeat in exon 1 of the huntingtin (*HTT*) gene. It is marked by progressive degeneration of neurons particularly in the striatum [4, 13]. Anyone who inherits an expanded CAG TNR in the mutant (m)*HTT* gene in the full penetrance range (>39 repeats) will develop the disease, with the length of the CAG inversely correlating with the severity and age of onset of the disease [13, 14]. Gene silencing experiments in mouse models have shown that when the expression of m*HTT* is reduced symptoms improve [15].

SBMA is a disease caused by an expanded CAG repeat present in exon 1b of the androgen receptor (*AR*). It is an X-linked sex-limited recessive adult-onset neurodegenerative disorder that involves the degeneration of the spinal and bulbar motor neurons and dorsal root ganglia [16, 17]. As with HD, the age of a patient at the time of disease onset correlates negatively with the length of the CAG repeat in the disease allele [18].

While there are many approaches to reduce mHTT currently in clinical trials, one of the earliest, that of using antisense oligonucleotides (ASOs) to reduce mHTT mRNA in HD mouse models [19], could not be replicated in clinical trials in humans (www.businesswire.com/news/home/20210322005754/en/Genentech-Provides-Update-on-Tominersen-Program-in-Manifest-Huntingtons-Disease., https://ir.wavelifesciences.com/news-releases/news-release-details/wave-life-sciences-announces-topline-data-and-addition-higher). While the reason for the trials failures is not yet published, they may have failed because the ASOs used in patients were not selective enough for mHTT and may have caused concomitant reduction of normal HTT that is critical for cell survival [20–22]. To design an effective treatment for HD, it is therefore imperative that the mechanisms contributing to the disease are fully understood.

Since the discovery of the CAG TNR diseases the poly glutamine (polyQ) mutant protein has received the most attention as the likely disease-causing moiety [23, 24]. Only later was it realized that mutant CAG (mCAG) RNA could also contribute to disease pathology by forming hairpin (HP) structures [25, 26]. Multiple mechanisms have been discovered and proposed for how mCAG-mRNA may be toxic (see Ref. [27] for a review). They include mCAG-RNA forming nucleolar foci that sequester splicing factors such as muscleblind-like 1 (MBNL1) [28], possibly occurring in a process of phase separation [29], sequestration of nucleolin resulting in a decrease in rRNA levels, enhanced translation from mCAG-RNA [30, 31], changes in nuclear export [32], and the production of small peptides via RAN translation [33]. Toxicity of mCAG-mRNA can also arise through the mechanism of RNA interference (RNAi) [34, 35].

RNAi is a form of post-transcriptional regulation exerted by 19–25 nt long double stranded (ds) RNAs that negatively regulate gene expression at the mRNA level. The active guide strand is incorporated into the RNA-induced silencing complex (RISC) [36] and the inactive passenger strand is degraded [37]. Depending on the degree of complementarity between the guide strand and its target, the outcome of RNAi can either be target degradation (most often achieved by siRNAs with full complementarity to their target mRNA; [38]) or miRNA-like cleavage-independent translational repression [39]. miRNAs are transcribed in the nucleus as primary miRNA precursors [40] which are first processed by the Drosha/DGCR8 microprocessor complex into pre-miRNAs [41], and then exported from the nucleus to the cytoplasm [42]. Once in the cytoplasm, Dicer/TRBP processes them further [43, 44] and these mature dsRNA duplexes are then loaded onto Argonaute (Ago) proteins to form the RISC [36]. CAG repeats in mHTT can form HP structures with stem regions of incomplete complementarity (so called R-loops; [45]). These can be processed by Dicer resulting in 21–22 nt long sCAGs that enter the RISC and silence specific targets [35, 46, 47] and sCAGs are toxic to neurons through RNA interference (RNAi) [34, 35].

sCAGs contribute substantially to disease pathology because treatment of R6/2 HD mice with locked nucleic acid (LNA)–modified ASOs complementary to the sCAGs (LNA-CUG) which selectively bind and block sCAGs that act through RNAi produced a rapid and sustained improvement of motor deficits [48]. More recently it was demonstrated that short RNAs isolated from m*HTT* transgenic R6/2 mice or *post mortem* HD patient (but not normal) brains, when transfected into differentiated SH-SY5Y cells reduced viability [34]. Furthermore, small RNAs isolated from postmortem HD but not from normal control brains could induce HD-like symptoms in mice after injection into their brains [49]. Most importantly, a substantial amount of the symptoms could be ameliorated after treating the mice with LNA-CUG [49]. These data are highly significant in the light of our recent discovery that CAG-based siRNAs, when entering the RISC, become super toxic to cancer cells by targeting genes containing extended CUG TNRs required for cell survival [50]. This provides a new powerful cell death-inducing mechanism with potential relevance to CAG repeat diseases.

Recently, two reports provided strong evidence that in HD it is the length of the CAG TNRs rather than polyQ length that determines the age at onset of symptoms [51, 52]. The first study identified rare subjects with HD who had either a loss of interrupting CAA (which also codes for glutamine) nucleotides or a CAACAG-duplication allele [51]. The age at onset was consistently later for individuals with a CAACAG-duplication allele, even though this allele specifies four more glutamines than a CAA-loss allele. The second study reported that HTT (CAG)_40_-(CAA-CAG)-CCG-CCA-(CCG)_7_ (Ref sequence) versus mHTT (CAG)_40_-(CA**G**-CAG)-CCG-CC**G**-(CCG)_7_ (loss of inhibition [LOI] sequence) patients have a dramatically reduced onset of disease by 25 years [52]. Both studies came to the conclusion that the number of uninterrupted CAG repeats is a more significant contributor to age of onset of HD than polyQ length, which is not altered in these individuals. This again focused the attention on mutant CAG-RNA as a disease causing agent.

Using different RNA seq analyses and data sets from normal and HD brains we now demonstrate that CAG TNRs can barely be detected by RNA seq providing an explanation for why they have rarely been observed and hitherto not often considered to be relevant. We now show that genes with CUG repeats of 10 nts and longer are significantly downregulated in HD patient brains and in the striatum of a HD mouse model, consistent with them being targeted by CAG TNRs through RNAi.

Both short HPs mimicking mHTT and mAR kill cells through RNAi as the HPs are not toxic to cells lacking either Dicer or Ago2 expression. For both HPs we show that it is the length of uninterrupted CAG containing stems that determines their stability and toxicity. We generated short HP mimetics of the HTT Ref and the LOI sequences and demonstrate that the LOI HP forms a bipartite structure with a greatly extended CAG-containing double stranded stem compared to the Ref HP. Consequently, when transfected it is significantly more toxic to cells than the Ref HP. Using Ago pulldown combined with RNA seq we show that when overexpressed RISC-bound sCAGs can be quantified. The LOI HP gives rise to about four times more toxic sCAG of 21–22 nt in length to enter the RISC than the Ref HP. Our data provide an explanation for why patients carrying the LOI *mHTT* allele which differs from the Ref allele by only two CAGs have a disease age at onset 25 year earlier. We suggest that targeting sCAGs rather than the entire mCAG-RNA could be a relevant approach to treating HD without the need to selectively target mutant alleles in the different CAG TNR diseases.

## Materials and methods

### Cell lines and tissue culture and reagents

All cells were grown in an atmosphere of 5% carbon dioxide (CO_2_) at 37 °C. Unless indicated otherwise base media were supplemented with 10% heat-inactivated fetal bovine serum (Serum Plus II; Sigma-Aldrich) and 1% penicillin/streptomycin and L-Glutamine (Mediatech Inc.). Cells were dissociated with 0.25% (w/v) Trypsin—0.53 mM EDTA solution (Mediatech Inc.). 293T parental and Dicer knock out cells (clone 4–25, provided by Dr. Bryan Cullen, Duke University) (RRID:CVCL_0063) were cultured in DMEM (Cellgro). The HCT116 Ago 1/2/3 k.o. cells [53] were provided by David Corey (UT Southwestern). HCT116 Dicer k.o. cells were purchased from the Korean Collection for Type Cultures (KCTC, clone #43, cat #HC19023) and cultured in McCoy 5 A medium. Neuroblastoma cell line NB7 [54] was cultured in RPMI1640. Ago2 ko 293T cells (provided by Dr. Klaas Mulder, Radboud Institute for Molecular Life Sciences, Nijmegen, the Netherlands) and HeLa wt and Ago2 ko cells [55] (provided by Dr. Sarah Gallois-Montbrun, Université Paris Descartes, Paris, France), were all cultured in DMEM (Cellgro). Lipofectamine RNAiMAX was from ThermoFisher Scientific (#13778150).

### Western blot analysis

Primary antibodies for Western blot: anti-β-actin antibody (Santa Cruz #sc-47778, RRID:AB_626632), anti-human AGO2 (Abcam #AB186733, RRID:AB_2713978). anti-human AGO1 (D84G10, Cell Signaling, #5053) and anti-human DICER Rabbit mAb (D38E7, Cell Signaling #5362). Secondary antibodies for Western blot: Goat anti-rabbit-IgG-HRP (Southern Biotech #SB-4030-05, RRID:AB_2687483). Western blot analysis was performed as recently described [56]. All uncropped blots are shown in Fig. S2.

### Transfection with short oligonucleotides and HPs

For transfection of cancer cells with siRNAs or hairpins Lipofectamine RNAiMax was used at a concentration optimized for each cell line, following the instructions of the vendor. Cell lines were transfected during plating (reverse transfection). For an IncuCyte experiment 50 μl transfection mix with RNAiMAX and 2.5 to 25 nM siRNAs were plated and cells were added in 200 μl of antibiotics-free medium. During growth curve acquisitions the medium was not exchanged to avoid perturbations. For the Ago pull down experiment with NB7 cells a large scale transfection preparation was set up using forward transfection. 5 million cells were plated and the next day 20 ml of fresh antibiotics-free medium was added in 5 ml of transfection mix. Cells were harvested, washed with PBS and cell pellets shock frozen, and stored at -80 °C until use. All individual RNA oligonucleotides were ordered from Integrated DNA Technologies (IDT).

Control siRNA: siNT1 (sense: mUmGrGrUrUrUrArCrArUrGrUrCrGrArCrUrArATT; antisense rUrUrArGrUrCrGrArCrArUrGrUrArArArCrCrAAA) non-targeting in mammalian cells.

RNA-hairpins with the following sequences were utilized in this study:

**Ctr HP (CAG)**_**7**_ **:**

rCrArGrCrArGrCrArGrCrArGrCrArGrCrArGrCrArG;

**Ctr HP (CAG)**_**12**_**:** rCrArGrCrArGrCrArGrCrArGrCrArGrCrArGrCrArGrCrArGrCrArGrCrArGrCrArGrCrArG;

**Ctr HP (CAG)**_**21**_**:** rArGrCrArGrCrArGrCrArGrCrArGrCrArGrCrArGrCrArGrCrArGrCrArGrCrArGrCrArGrCrArGrCrArGrCrArGrCrArGrCrArGrCrArGrCrArGrCrArGrCrArGrCrArG;

**Ctr HP (CAG)**_**29**_**:** rCrArGrCrArGrCrArGrCrArGrCrArGrCrArGrCrArGrCrArGrCrArGrCrArGrCrArGrCrArGrCrArGrCrArGrCrArGrCrArGrCrArGrCrArGrCrArGrCrArGrCrArGrCrArGrCrArGrCrArGrCrArGrCrArGrCrArGrCrArGrCrArG;

**Ctr HP (CAG)**_**40**_**:** CrArGrCrArGrCrArGrCrArGrCrArGrCrArGrCrArGrCrArGrCrArGrCrArGrCrArGrCrArGrCrArGrCrArGrCrArGrCrArGrCrArGrCrArGrCrArGrCrArGrCrArGrCrArGrCrArGrCrArGrCrArGrCrArGrCrArGrCrArGrCrArGrCrArGrCrArGrCrArGrCrArGrCrArGrCrArGrCrArGrCrArGrCrArGrCrArGrCrArG;

**AR-HP 3-9**: rUrGrCrUrGrCrUrGrCrUrGrCrArGrCrArGrCrArGrCrArGrCrArGrCrArGrCrArGrCrArGrCrArGrCrArA;

**AR-HP 3-17**: rUrGrCrUrGrCrUrGrCrUrGrCrArGrCrArGrCrArGrCrArGrCrArGrCrArGrCrArGrCrArGrCrArGrCrArGrCrArGrCrArGrCrArGrCrArGrCrArGrCrArGrCrArGrCrArA;

**Ref-HP:** rCrArGrCrArGrCrArGrCrArGrCrArGrCrArGrCrArGrCrArGrCrArGrCrArGrCrArGrCrArGrCrArGrCrArGrCrArGrCrArGrCrArGrCrArArCrArGrCrCrGrCrCrArCrCrGrCrCrGrCrCrGrCrCrGrCrCrGrCrCrGrCrCrGrCrCrU;

**LOI-HP**: rCrArGrCrArGrCrArGrCrArGrCrArGrCrArGrCrArGrCrArGrCrArGrCrArGrCrArGrCrArGrCrArGrCrArGrCrArGrCrArGrCrArGrCrArGrCrArGrCrCrGrCrCrGrCrCrGrCrCrGrCrCrGrCrCrGrCrCrGrCrCrGrCrCrGrCrCrU.

### Ago Pull-Down and subsequent small RNA seq

R6/2 mice and their wild-type littermates (C57/BL6J) were taken from a colony established at the University of Cambridge as previously described [57, 58]. Tail snips were taken at 3 weeks of age for genotyping and CAG repeat sizing (Laragen, Los Angeles, CA). CAG repeat lengths were measured by GeneMapper software (Life Technologies, NY). R6/2_250 and R6/2_450 mice had a mean CAG repeat length of ~250 ± 1 (*n* = 3) or ~450 ± 7-10 (*n* = 2). All experiments were conducted under the authority of the United Kingdom Animals (Scientific Procedures) Act 1986 Amendment Regulations 2012, and with the approval of the University of Cambridge Animal Welfare and Ethical Review Body. Mouse brain lysates were prepared (three wild-type [18–19 weeks old], three 250CAG repeat [19 weeks old], and two 450CAG repeat [85 and 90 weeks old]) by first chopping 100-250 mg brain (striatum) tissue with a clean razor blade and then using a Dounce homogenizer containing 1 ml NET lysis buffer/100 mg of tissue (TBS, 5 mM EDTA, 0.5% NP40, 10% Glycerol, 1 mM NaF, 1 mM AEBSF). The tissue was homogenized by passing the pestle up and down the cylinder 100 times while keeping the homogenizer cool on ice. Cell or tissue lysates were then incubated on ice for 15 min, vortexed, and then centrifuged at 20,000 g for 20 min. The lysates were then transferred to siliconized microcentrifuge tubes (low-binding, Eppendorf #022431021), small RISC-bound RNAs were pulled down using Flag-GST-T6B peptide [59] and anti-Flag M2 Magnetic beads (Sigma #M8823), a library was prepared and then sequenced on an Illumina Hi-Seq 4000 exactly as previously described [60]. RNA seq data can be accessed at GSE201691 and GSE201692.

Sequences used for small RNA library preparation:

19 nt RNA size marker: rCrGrUrArCrGrCrGrGrGrUrUrUrArArArCrGrA;

35 nt RNA size marker: rCrUrCrArUrCrUrUrGrGrUrCrGrUrArCrGrCrGrGrArArUrArGrUrUrUrArArArCrUrGrU;

To identify the reads derived from the HTT HPs, we used regular expressions within Perl to extract all reads that contained one of the following 19 nt long sequences: group 1: CAGCAGCAGCAGCAGCAGC, AGCAGCAGCAGCAGCAGCA, GCAGCAGCAGCAGCAGCAG; group 2: CCGCCGCCGCCGCCGCCGC, CGCCGCCGCCGCCGCCGCC, GCCGCCGCCGCCGCCGCCG. Reads were summed up in the two groups in all samples as well as all remaining reads were summed up as group 3.

### Small RNA seq of short RNA oligonucleotides

Small RNA libraries for the 19 nt and 35 nt RNA size marker (sequences above) as well as for (CAG)_7_ and (CAG)_12_ were prepared as described above for library post Ago pull down. In each case, 10 pmol RNA was radiolabeled as described [61] before proceeding for library preparation. For Set 1 (Fig. 5A), post 3' ligation with adenylated adapter, the 19 nt RNA was combined with 35 nt and (CAG)_7_ RNA was combined with (CAG)_12_ and then 5' ligation was performed individually for the two combined samples. For Set 2 (Fig. 5A), all four RNA samples were combined post 3' ligation. After reverse transcription, cDNA for Set 1 was amplified using two different 3' PCR primers for the two combined samples and for Set 2, only one 3' PCR primer was used. Post sequencing on Illumina Hi-Seq 4000, the reads for Set 1 were first separated by Illumina based on 3' PCR primers and then both for Set 1 and 2 using the barcode on 3' adenylated adapters. RNA seq data can be accessed at GSE201694.

### Monitoring growth over time and quantification of cell death

To monitor cell growth over time, cells were seeded between 1000 and 4000 per well in a 96-well plate in triplicates. The plate was then scanned using the IncuCyte ZOOM live cell imaging system (Essen BioScience). Images were captured at regular intervals, at the indicated time points, using a 10x objective. Cell confluence was calculated using the IncuCyte ZOOM software (version 2015A). A viability assay that measures the level of ATP within cells was done in 96-well plates. Briefly, 96 h post reverse transfection with siRNAs or HPs, media in each well was replaced with 50 μl fresh medium and 50 μl of Cell Titer-Glo reagent (Promega #G7570) was added. The plates were covered with aluminum foil and shaken for 5 min and then incubated for 10 min at room temperature before the luminescence was read on a BioTek Cytation 5.

### RNA secondary structure predictions and binding energy calculations

To determine the folding and binding energies of HTT or AR HPs, we used RNAfold [62] (at http://rna.tbi.univie.ac.at/cgi-bin/RNAWebSuite/RNAfold.cgi) with the following settings: (1) Fold algorithms and basic options: minimum free energy (MFE) and partition function, avoid isolated base pairs, dangling energies on both sides of a helix in any case; (2) Energy parameters: RNA parameters (Turner model, 1999); After conversion of SHAPE reactivities, apply pseudo energies to: Stacked pairs; slope (m): 1.9; intercept (b): 0.7. We chose as output options: interactive RNA secondary structure plot. For each RNA the structure with the lowest ΔG was used. We either subjected the TNR containing regions of wt*HTT* and its mutants with 15 extra nucleotides added to the 5' and the 3' end or the mHTT and mAR mimicking short HPs as well as pure CAG TNR containing oligonucleotides to the analysis.

### Data analyses

**Fig. 1:**
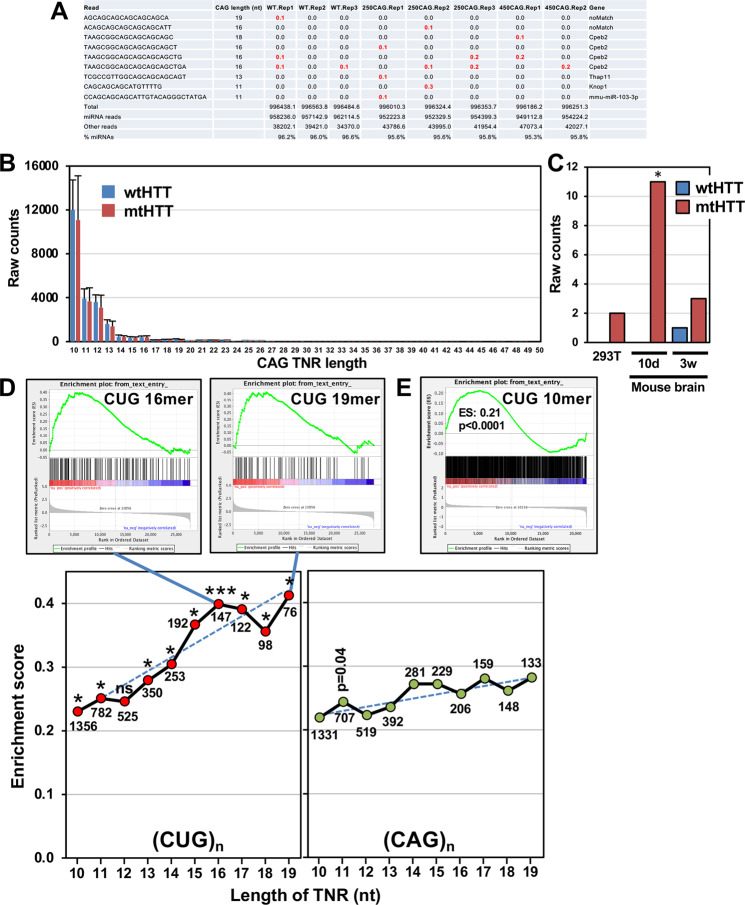
Enrichment of CUG repeat containing mRNAs of genes downregulated in HD patient brains and a HD mouse model. **A** A list of all reads with (CAG)_*n*_ of 8 nt or longer detected in an Ago pulldown and RNA sequencing experiment of mouse brains (either wt, 250CAG or 450CAG mice). Conditions in which a few reads were found are labeled in red numbers. **B** Comparison of the number of raw reads derived from genes containing CAG repeats of different lengths (10–50 nt) in 293T cells infected with a lentivirus coding for either *HTT* exon 1 with 54 CAG nt (Q18, wt) or with 198 nts (Q66, mt). Data of triplicate samples were extracted from GSE78928. **C** Number of individual reads comprised of pure (CAG)_*n*_, (AGC)_n_ or (GCA)_n_ of 50 nts in either 293T cells infected with lentiviral wtHTT or mtHTT or in mouse brains 10 days or 3 weeks after injection with either AAV-Q18 or AAV-Q66. Shown are the sums of read counts of three replicates for each condition. *Significance of Fisher’s exact test <0.05. **D** A list of 27785 genes deregulated in age matched postmortem brains of 20 HD patients compared to 49 normal brains, ranked from highest to lowest downregulation was subjected to GSEA using lists of all human genes that contain (CUG)_n_ or (CAG)_*n*_ repeat sequences of different lengths (10–19 nts). RNA seq data were extracted at GSE64810. Enrichment scores and normalized *p*-values are given. *p-value < 0.05; ***<0.0001. Numbers next to data points indicate the number of TNR containing genes in each analysis. Top: two examples of the most significantly enriched gene lists that contain (CUG)_n_ of at least 16 nts and 19 nts in length. **E** A list of genes deregulated in the striatum of Q111 HD mice compared to Q20 HD mice, ranked from highest to lowest downregulation was subjected to GSEA using lists of all mouse genes that contain (CUG)_n_ repeat sequences of 10 nts or longer. RNA seq data were extracted at GSE50379. Enrichment scores and normalized *p*-values are given.

For the analysis of sCAGs in the Ago pull down RNA seq analysis in Fig. 1A SPOROS output A_normCounts was generated as described [63]. This file includes BLAST search results for murine miRNAs and all RNA classes. This information was used to calculate the percent miRNA content for each sample. All reads with uninterrupted CAG repeats of 11 nts or longer were identified and listed.

For the analysis in Fig. 1B we used an RNA seq data (50 nucleotides read length) set of 293T cells infected with lentiviral vectors expressing exon 1 of wild-type HTT (wtHTT, 18 polyQ repeats) or mutant HTT (mHTT 66Q, 66 polyQ repeats)—all in triplicates [64]. The data were obtained from GEO, accession number GSE78928. To identify all reads that contained CAG repeats of lengths ranging from 10 to 50 nts, we generated 40 files in which we isolated a CAG repeats (10,11,12…..or 50 nts in lengths) in each individual read from the preceding and trailing nucleotides and then counted the number of reads in each file. Every read was only counted once in the group with the longest repeat length it appeared. The average read numbers that contain different lengths of CAG repeats were plotted with Standard Deviation in Fig. 1B.

For the analysis in Fig. 1C, the same data set was used in addition to triplicate RNA seq data sets generated from brains of mice infected with adeno associated viral vectors expressing exon 1 of wtHTT or mutant HTT [64] either 10 days or 3 weeks after injection of viruses. In these cases all 50mer reads comprised of pure CAG, AGC or GCA repeats were counted.

To perform the analysis in Fig. 1D, we first generated lists of all human genes that contain either a CAG or a CUG repeat sequence of 10, 11, 12….19 nts nucleotides in length or longer in their mRNA. To this end all 5'UTRs, ORFs and 3'UTRs were extracted from the *Homo sapiens* (GRCh38.p7) gene dataset of the Ensembl database using the Ensembl Biomart data mining tool. To perform the analysis in Fig. 1E, we first generated lists of all murine genes that contain a CUG repeat sequence of 10 nts or longer in their mRNA. To this end all 5'UTRs, ORFs and 3'UTRs were extracted from the *Mus musculus* (GRCm39) gene dataset of the Ensembl database using the Ensembl Biomart data mining tool. For each gene, only the longest deposited 5'UTR, ORF, or 3'UTR was stitched together. Custom perl scripts were used to identify whether each mRNA contained an identical match to a particular repeat sequence.

GSEA was performed using the GSEA v2.2.4 software from the Broad Institute (www.http://software.broadinstitute.org/gsea); 1000 permutations were used. 20 lists (see above) with the genes containing genes with the different CAG or CUG lengths were used. They were set as custom gene sets to determine enrichment of genes in downregulated genes from an RNA-seq data set comparing expression of genes between brains of 49 normal brains and 20 brains from HD patients as described [65]. The human data were retrieved from GSE64810, the mouse data from GSE50379. Log(Fold Change) was used as the ranking metric. *p*-values below 0.05 were considered significantly enriched.

For the analysis shown in Fig. S1 gene array data sets on 293T, HeLa and human brains were downloaded from GEO (accession numbers: GSE171397 and GSE209928, and GSE64810). The data of all coding genes from untreated cells or control brains were extracted and each sample was normalized to one million reads. All human genes containing (CUG)_n_, (UGC)_n_, or (GCU)_*n*_ repeats of 10 or more nucleotides in length were highlighted as well as all genes that are part of the list of critical survival genes available at DepMap.org (version 22Q2). We downloaded all 2165 genes that were shown to be critical of survival of any of the 1840 different cell lines tested. Percent expression of these genes was calculated and pie charts were generated in Excel. Venn diagrams of all potential target genes in the three data sets with normalized expression signals of >100 were generated using http://bioinformatics.psb.ugent.be/webtools/Venn/ and http://www.biovenn.nl (to obtain the correct size proportional circles).

### Statistical analyses

Two-way analysis of variances (ANOVA) was performed using the Stata 14 software to compare treatment effects over the course of the experiment for the varying cell types. The Fishers exact test for Fig. 1C was done by using the online tool at https://www.socscistatistics.com/tests/fisher/default2.aspx. All other statistical analyses were conducted in Stata 14 (RRID:SCR_012763) or R 3.3.1 in Rstudio (RRID:SCR_000432).

## Results

### Evidence of silencing of CUG TNR containing genes in the brains of HD patients and HD mice

Even though RNAi active sCAGs of 21 nt in length form and can be detected specifically in HD patients using either Northern blotting or sequencing after polyadenylating and cloning them into a sequencing vector, the amount of sCAG was found to be very difficult to quantify by RNA seq analysis [34]. We have made similar observations. In an RNA seq analysis of RISC-bound small RNAs in brains of R/6 mice with 250 or 450 CAG long TNRs [66] we did not find a single read with a CAG TNR >19 nt and all CAG TNR containing reads were either detected at background levels or were derived from other genes (red bold numbers in Fig. 1A). This was also apparent when the RNA seq data from another study were examined [64]. That study employed expression of exon 1 of *HTT* containing either a wild-type (wt) length of CAG TNRs (18Q, 54 nts) or a mutant length (66Q, 198 nts). It was intriguing that in a large RNA seq analysis no increase in (CAG)_*n*_-containing reads between 10 and 50 nt in length was detected in 293T cells infected with a lentiviral mt*HTT* when compared to cells infected with lentiviral wt*HTT* (Fig. 1B). In addition, even the reads of short (CAG)_*n*_ containing genes were of very low abundance. A similar finding was made when the number of reads with pure (CAG)_*n*_ were counted in an RNA seq data set of mouse brains infected with an adeno associated virus (AAV) expressing either wt or mt*HTT* (Fig. 1C). Only 11 reads with 50 nt long CAG, AGC or GCA repeats were detected in these mice 10 days after infection, with even fewer reads detectable at 3 weeks after infection. Not a single pure (CAG)_*n*_ containing read of 19 nt or longer was detected in any of the three replicates of the small RNA seq samples or with an RNA immunoprecipitation sequencing assay (data not shown). The reason for the difficulties of detecting CAG TNR containing RNAs by RNA seq is not known but is likely due to the repetitive nature of these RNA species.

We therefore decided to test whether in HD patients we could find indirect evidence of the expression of CAG TNR containing RNAs. Assuming that they act through RNAi we would expect to find a downregulation of genes containing the target sequence of a CAG containing small RNA: CUG trinucleotide repeats [(CUG)_*n*_]. We previously provided evidence with in vitro transfected cells that a CAG derived siRNA of 19 nts caused a significant downregulation of genes that contained CUG TNRs of 19 nt or longer [50]. We chose a large RNA seq data set from a study that compared gene expression between 49 normal and 20 HD patient brains [65] to perform gene set enrichment analyses (GSEA) with ten different lists of genes that contain CUG repeats of 10 nt or longer, 11 nt or longer, etc. up to 19 nt or longer assuming various lengths of complementarity between the sCAGs and (CUG)_n_-containing targets. Enrichment scores increased with longer CUG TNRs and all but one was statistically significant (Fig. 1D, bottom left). This suggests that CAG TNR can target a variety of genes with different lengths of CUG TNRs. It appears that the most significant downregulation was found with genes containing a CUG TNR of 16 nts and 19 nts (GSEA graphs on top of Fig. 1D). In contrast, the increase in enrichment with longer TNR length was much less pronounced in genes containing CAG TNRs and all but one did not reach statistical significance even though the number of genes containing either CAG or CUG TNRs for each TNR length was comparable (numbers in bottom panels in Fig. 1D). Similar results were obtained by analyzing a gene array data set of control (Hdh(Q20/Q20)) and mutant HD (Hdh(Q111/Q111)) mice [67]. An enrichment of (CUG)_n_ (10 nt or longer) containing genes was found in the genes downregulated in striatum of the Q111 versus the Q20 mice (Fig. 1E). These data suggest that in HD patient brains and a HD mouse model there is selective pressure on downregulation of CUG TNR containing genes consistent with the interpretation that they could be targeted by CAG TNR containing short RNAs through RNAi.

### The length of uninterrupted stem regions in CAG TNR containing HD derived hairpins correlates with disease severity and inversely correlates with disease onset

**Fig. 2:**
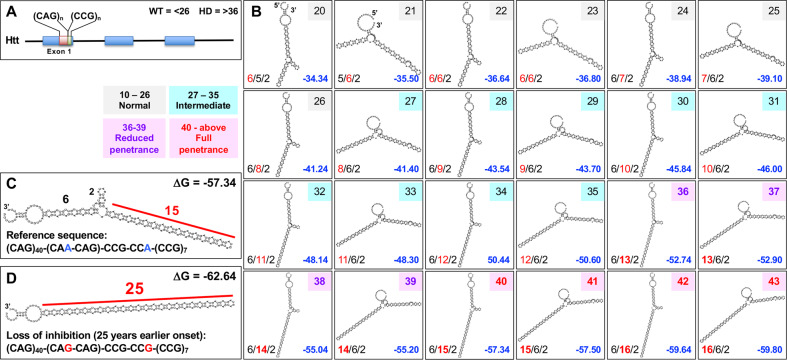
Folding energies and stem lengths of folded CAG TNRs found in HD patient RNAs correlate with disease severity and/or onset. **A** Location of the CAG TNR in exon 1 of the *HTT* gene [71]. **B** Predicted secondary structure of the section of the HTT mRNAs containing the CAG TNRs (plus 15 nt at either end) at different lengths found in normal, intermediate, reduced, or full penetrance patients. The length of the double stranded CAG TNRs in the stem regions in each HP is given with the longest contiguous one in red. ΔG values (in kcal/mol) are given in blue. **C**, **D** Predicted secondary structure of the section of *HTT* either containing the predominant mutant length of 40 CAG TNRs (the reference sequence) (**C**) or the rare mutant length of 42 CAG TNRs (the loss of inhibition/LOI mutant) (**D**) (plus 15 nts on either side). Secondary structures were predicted using RNAfold. Longest uninterrupted double stranded CAG repeats are labeled with repeat numbers in red. Folding energies are given.

Patients develop HD when the length of the CAG expansion in the *HTT* gene exceeds 36 TNRs (Fig. 2A) [14]. The R-loop structure that is formed by the CAG TNRs present in *HTT* can be predicted to fold into extended stems interrupted by loop regions (Fig. 2B). It has been shown that such stem containing HPs are substrates for Dicer [35]. We therefore predicted that the longer the stem that forms in mutant HTT (mHTT) is and the lower the binding energy, the more sCAG will form as these structures will be better substrates for Dicer. To test this hypothesis in a simulation, we performed RNA folding experiments of the section in HTT containing an increasing length of CAG TNR stretches (Fig. 2B). The longest stem of 16 repeats was predicted to form in the RNAs with the longest uninterrupted CAG TNR. At the same time the stability of these structures also increased (as shown by the decreasing binding energies) with an increased TNR length. The increase of stem lengths from 6 to 16 CAG TNRs correlates with a worsening in HD disease scores [13].

An open question remains as to how extending the uninterrupted CAG TNR length from 40 to 42 in the HTT LOI mutant by adding just two point mutations (Fig. 2C, D) could result in a dramatic reduction in disease onset by 25 years [52]. We predicted that these minor changes may affect the folding of the HPs in a way that would allow them to form more stable structures with strongly extended uninterrupted CAG TNR containing stem regions. When we compared the predicted secondary structure of the *HTT* reference sequence with that of the LOI mutant, we found a profound shift from a tripartite stem structure disrupted by a loop region and a longest stem of 15 CAG TNRs to a more stable bipartite structure forming one long stem region of 25 CAG TNRs, by far the longest uninterrupted CAG TNR containing stem detected in any RNA folding analysis of mHTT with the lowest binding energy (Fig. 2C, D). The extended CAG repeat containing stem region in the LOI allele could be a better substrate for Dicer and result in generation of an increased amount of sCAGs.

### Short oligonucleotide mimetics of the reference and LOI *HTT* mutants have different levels of toxicity on cells through RNAi

**Fig. 3:**
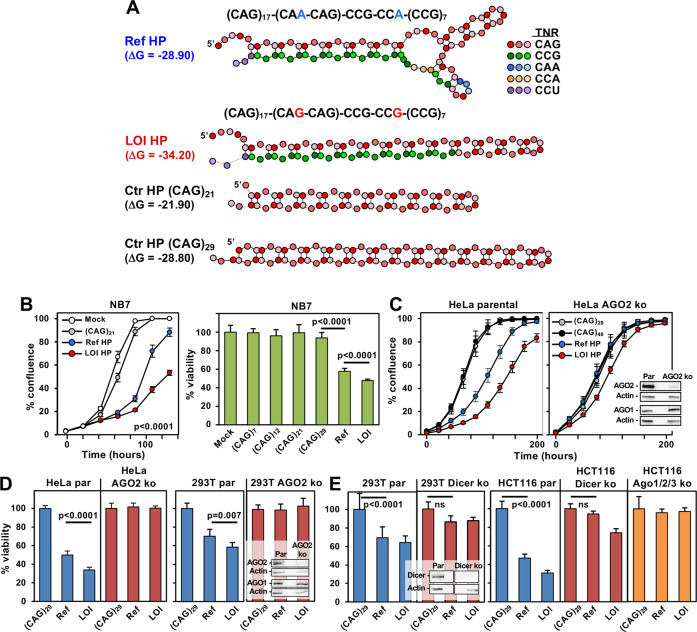
A LOI mutant HP mimetic is more toxic through RNAi than the reference HP. **A** Secondary structures of the synthesized RNA HPs including two control HPs comprised of pure CAG repeats. Different TNRs are shown in different colors. Binding energies are given. **B**, *left*, change in confluency over time of NB7 cells transfected with three of the HPs at 2.5 nM. **B***, right*, viability (ATP assay) of NB7 cells 4 days after transfection with the different HPs (1 nM). Fold change is shown relative to lipid treated cells. Two-way ANOVA is shown. **C** Change in confluency over time of HeLa parental or AGO2 k.o. cells transfected with four of the HPs at 25 nM. **D***, left panel*, viability of HeLa parental or AGO2 k.o. cells 4 days after transfection with the different HPs (25 nM). **D**, *right panel*, **E** Viability of indicated cells 4 days after transfection with the different HPs (10 nM for 293T cells, 2.5 nM for HCT116 cells). Fold change is shown relative to (CAG)_29_ control. Average of 3–6 replicates are shown −/+SE (**B**, left panel, **C**) and mean of 3-6 replicates are shown −/+SD (**B**, right panel, **D**, **E**). par = parental. *P*-values (Student’s *t*-test) are shown in **B**, right panel, **D** and **E**. ns, not significant. Inserts show Western blot analyses of the parental and corresponding mutant cell lines.

It was previously shown that the overall structural architecture of the triplet repeat region in four HTT transcripts that differed only by the length of the uninterrupted CAG TNR was very similar [35, 68]. We therefore predicted that a HP with shorter CAG repeats that can be easily synthesized and transfected would be a good mimetic of the overall structure formed by CAG repeats, and that structures with longer repeats would be even more toxic. We designed short HP models of the Ref and the LOI mHTT structures (Fig. 3A). As with the longer version, the short mimetics of these two variants had different binding energies and stem regions of different lengths. Single stranded pure CAG TNR containing oligonucleotides were used as a control. According to previous studies they were also expected to fold into a stem through the formation of R-loops [45]. To determine whether these HPs would affect cell viability differently, we transfected them into the neuroblastoma cell line NB7 [54]. Both the Ref and the LOI mutant slowed growth more than the (CAG)_21_ control HP (Fig. 3B, left panel). Interestingly, the LOI HP was significantly more toxic to the cells than the Ref HP. This was confirmed by viability assays which also included four pure (CAG)_*n*_ containing control hairpins. In contrast to the HD derived HPs, none of these (CAG)_*n*_ containing ones were toxic to the cells (Fig. 3B, right panel).

To determine whether the toxicity exerted by the HD derived HPs involved RNAi, we tested the two mutant HTT HPs in HeLa cells with a deletion of Ago2 (Fig. 3C). These Ago2 knockout cells were completely resistant to cell growth inhibition by the Ref HP and highly resistant to the effects of the LOI HP. In this experiment even a pure CAG containing HP of 40 CAG repeats had no activity. These data suggested that the observed toxicity was dependent on a functional RISC. This was also confirmed in viability assays (Fig. 3D). In neither HeLa nor 293T cells deficient in Ago2 expression did either of the two HD derived HPs show toxicity. Both 293T and HeLa cells express a substantial amount of genes (~7.5%) that contain CUG repeats of at least 10 nt in length (Fig. S1A, B) many of which are substantially expressed in both cell lines (Fig. S1A, B, D). Interestingly, 60% of the top ten most highly expressed (CUG)_*n*_ containing genes were critical survival genes (shown in red in Fig. S1A, B). Human brains also expressed about the same amount of (CUG)_*n*_ containing genes and two of the top ten most highly expressed ones were also in the top ten in the two cell lines (Fig. S1C). A substantial number of such genes were expressed in all three data sets (Fig. S1E).

A number of reports have demonstrated that (CAG)_*n*_ containing HPs are good substrates for Dicer [35, 45–47]. We therefore predicted that the two toxic HD derived HPs would not be toxic to cells deficient in Dicer expression. Indeed, the two HD derived HPs which were toxic to 293T parent cells did not significantly kill 293T Dicer ko cells (Fig. 3E, left two panels), however, a minor reduction in cell viability was still detected. To test whether any residual Dicer expression we detected by Western blotting on longer exposure in 293T Dicer ko cells (not shown), could have affected the results, we transfected the HCT116 cells which were shown to tolerate a complete biallelic deletion of Dicer [69] (Fig. 3E, right three panels). While the Ref HP was not toxic to these Dicer ko cells, the LOI HP still appeared to affect cell viability. It is possible however, that this was due to some loading of HP sequences into the RISC without the help of Dicer because cells deficient for AGO1, 2 and 3 were completely resistant to the toxicity of the two HD derived HPs (Fig. 3E, far right panel). These data also exclude that toxicity exerted by the HPs was due to binding of the HPs to other RNA binding proteins such as muscleblind 1 (MBNL1) [70].

### The toxicity of CAG TNR hairpin mimetic of mutant androgen receptor depends on the length of the CAG repeat containing stem

**Fig. 4:**
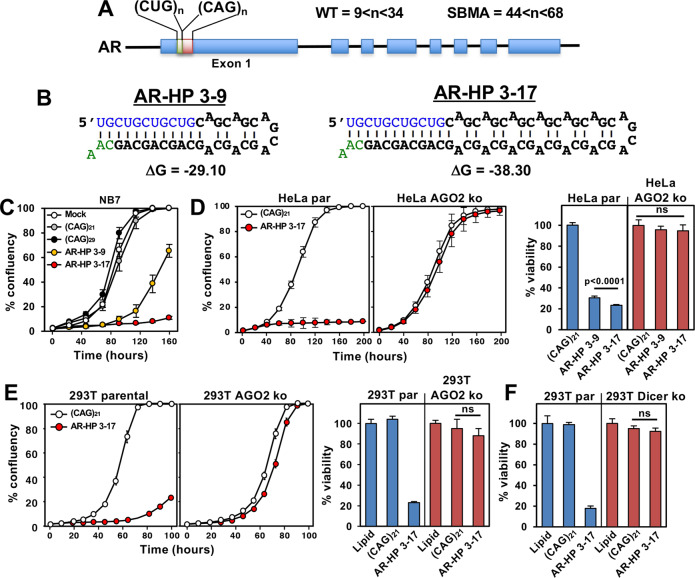
An AR CAG TNR based HP with longer CAG repeats more effectively kills cells through RNAi. **A** Location of the CAG repeat region in the *AR* gene. The mean length of the CAG repeat in the *AR* gene in different SBMA patient populations ranges between 44 and 48. The maximum repeat length is around 68, whereas the normal length is between 9 and 34 [3, 17, 75]. **B** Structure of the HP mimetics used. ΔG value (in kcal/mol) is given. **C**, **D**, *left panel*, change in confluency over time of NB7, HeLa parental or HeLa AGO2 k.o. cells transfected with 2.5 nM (for NB7 cells) or 10 nM (for HeLa cells) of different HPs. **D**, right panel, viability of indicated cells 4 days after transfection with different HPs (10 nM). Fold change is shown relative to (CAG)_21_ control. **E**, *left panel*, change in confluency over time of 293T parental or AGO2 k.o. cells transfected with 25 nM of either (CAG)_21_ or 3–17. **E**, *right panel*, **F**, viability of indicated cells 3 days (**E**) or 4 days (**F**) after transfection with 25 nM (**E**) or 10 nM (**F**) of either (CAG)_21_ or 3–17. Fold change is shown relative to lipid control. *p* = values (Student’s *t*-test) are shown (**D**, right panel, **E**, right panel, **F**). Average of 3-6 replicates are shown −/+SE (**C**, **D**, left panel, **E**, left panel) and mean of 3–6 replicates are shown −/+SD (**D**, right panel, **E**).

The idea that a more stable HP makes it more toxic was also proposed for HPs that were predicted to form in the CAG TNR expansion present in *AR* causing SBMA [68]. It was shown that the stability of both HTT and AR HP structures in vitro is affected by neighboring repeat regions [68]. In the *HTT* locus, there is a polymorphic CCG tract that is 12 bp downstream of the expansion-prone (CAG)_*n*_ (Fig. 2A). Similarly, the *AR* locus contains a (CTG)_3_(CAG)_*n*_ sequence (Fig. 4A) with a monomorphic (CAG)_6_ tract 18 bp downstream [3]. We predicted that this stabilized structure in mAR may also result in it being a better substrate for Dicer and that this structure would be highly toxic to cells via RNAi. We also predicted that a longer CAG repeat containing stem region in the HP would result in production of a higher amount of sCAG and hence greater toxicity. To test this hypothesis, we synthesized two *AR* gene derived short HP mimetics with a CAG TNR-containing stem stabilized by the authentic CAG/CUG TNR clamp at its base (Fig. 4B). One contained 3 CUG repeats and 9 CAG repeats (AR-HP 3-9) and the other 3 CUG and 17 CAG repeats (AR-HP 3-17). The 3-17 HP was predicted to form a more stable structure than the 3–9 HP. When transfected into NB7 cells the 3-17 HP was more toxic than that 3–9 HP (Fig. 4C). It was also more toxic than even the HD derived LOI HP likely due to forming a more stable structure caused by its complete complementarity in the CAGCAGCAGCA:UGCUGCUGCUG clamp. Even the high toxicity of the 3-17 HP was due to RNAi as both HeLa and 293T cells lacking Ago2 expression were completely protected from this toxicity (Fig. 4D, E). Similar to the results obtained with the HD derived HPs the AR derived HP did not kill 293T cells deficient in Dicer expression (Fig. 4F). These data suggest that short HPs mimic the activity of the longer sequences found in either HD or SBMA patients and that a combination of the length of the CAG TNR-containing stem regions and their predicted folding energies affect the toxicity of the HP killing RNAi competent cells.

### The HD LOI hairpin produces more RISC-bound sCAGs than the reference hairpin

**Fig. 5:**
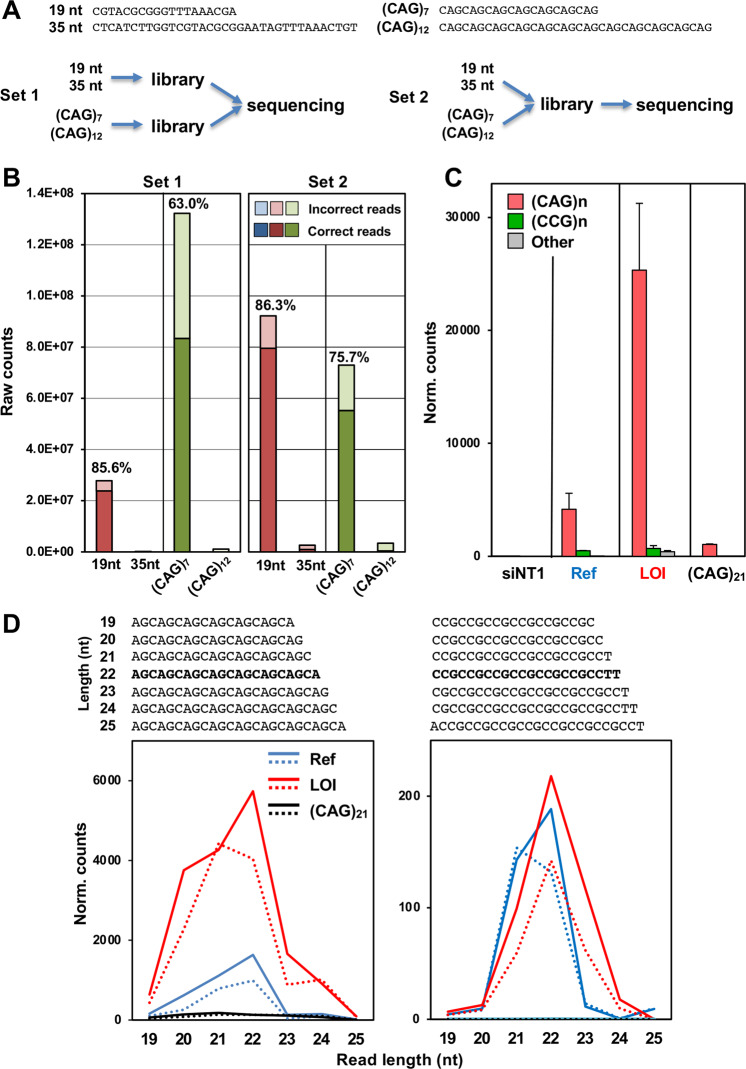
The LOI mutant HP results in a higher number of toxic sCAGs in the RISC of transfected cells than the Ref HP. **A** *Top*, the four sequences that were converted to libraries and sequenced by small RNA seq; *bottom*, schematics explaining two different sequencing approaches. For set 1 two libraries were prepared, one containing a 1:1 mixture of 10 pmol of size markers (19 and 35 nts long) and another containing a 1:1 mixture of 10 pmol of two CAG repeat containing RNAs (21 and 36 nt) and then together subjected to smRNA seq on an lllumina HiSeq 3000. For set 2 the four oligonucleotides were mixed 1:1:1:1 and the resulting library was subjected to sequencing. **B** Raw counts of the two libraries. Percent of reads that had the exact length and sequence of the RNA that was used are indicated. **C** Normalized read counts detected bound to the RISC in cells transfected with either 2.5 nM siNT1, (CAG)_21_, the Ref HP, or the LOI HP after 24 h (all as shown in **C**). Reads that contain only CAG TNR, CCG TNRs or other sequences are shown. Shown are the averages and the variance between the duplicate samples. **D** Sequences (top) and length distribution (bottom) of HP derived reads detected in the RISC of transfected cells in **D**. Solid and stippled lines of the same color represent the two duplicates.

We were wondering whether we would find a higher amount of RISC-bound sCAGs in cells transfected with the more stable and more toxic HD derived LOI HP compared to the Ref HP. However, our data and those by others [34] suggested that CAG TNRs are difficult to sequence on the Illumina platform. To test whether CAG TNR-containing RNAs could be sequenced at all, we generated sets of libraries for small RNA seq (Fig. 5A). In set 1 we used the Illumina platform to sequence two independent libraries: one derived from 10 pmol of two RNA size markers (19 and 35 nt, as nonrepetitive controls) and one that contained the same amount of two CAG TNR containing short RNAs (21 and 36 nt in length). We chose the 21 nt long CAG TNR sequence (CAG)_7_ as this is the length of short CAG repeat containing RNAs (sCAGs) that was shown to be associated with disease pathology in HD patients [34]. In set 2 we first mixed all four oligonucleotides and then sequenced the resulting library (Fig. 5A). This way CAG TNR containing oligonucleotides were in competition with the nonrepetitive size markers during all steps of library generation and sequencing. In none of the experiments were the larger oligonucleotides efficiently sequenced in this small RNA seq experiment. In set 1 (CAG)_7_ was more efficiently sequenced than the 19 nt marker. However, sequencing errors of (CAG)_7_ were much higher than seen with the control. Only 63% of all reads had the expected sequence and length (Fig. 5B, left). In set 2 sequencing of (CAG)_7_ was less efficient than that of the 19 nt marker suggesting that the CAG TNR-containing oligonucleotide was at a disadvantage compared to the nonrepetitive sequence (Fig. 5B, right). However, the results also suggested that it was possible to sequence sCAGs when they were present at high concentration.

We therefore decided to use RNA seq to analyze RISC-bound sCAGs in cells transfected with the HTT HP. The LOI HP contains a long stem with a mixture of (CAG)_*n*_ and (CCG)_*n*_ (Fig. 3A). We first transfected NB7 cells with 2.5 nM of these two HPs, the (CAG)_21_ and a nontargeting siRNA control (siNT1). We then performed an Ago pulldown as previously described [60] and sequenced the Ago bound small RNAs (Fig. 5C). We detected a significant number of pure CAG containing short RNAs in the cells transfected with the Ref HP, with only small amounts of CCG containing short RNAs. In the cells transfected with the LOI HP we found about four times more RISC-bound sCAGs but about the same small amount of short RNAs containing the CCG repeat sequence. These data are in line with a previous report showing that transcripts composed of CUG and CAG repeats are better Dicer substrates than those composed of CCG and CGG repeats [35]. The amount of CAG-containing short RNAs pulled down from cells transfected with the same amount of (CAG)_21_ was also small. These results suggest that (1) the LOI HP results in about four times more sCAGs bound to the RISC, consistent with the higher toxicity of this HP when compared to the Ref sequence, and (2) CAG-containing short RNAs are more efficiently loaded into the RISC than CCG containing sequences. The most abundant RISC-bound short RNAs were 21–22 nt in length (Fig. 5D) consistent with Dicer cleaving the HPs and in line with data from a previous analysis which found that Dicer cleavage of (CAG)_*n*_ results in 21–22 nt long sCAGs [35]. Interestingly, each length group only contained one defined species, with all CAG-containing RISC-bound short RNAs beginning with AGC and most of the abundant (CCG)_*n*_-containing short RNAs starting with CCG. The finding that the sequence and length of the most abundant RISC bound CAG TNR-containing short RNA is identical between the cells transfected with the LOI and the Ref HP suggests that it is the amount of these toxic sequences and not their sequence or length that distinguishes the LOI mutant from the Ref sequence. In summary, our data suggest that CAG repeat HPs derived from either HD or SBMA kill cells through RNAi after being processed by Dicer and that the HD LOI mutant is more toxic to cells than the reference sequence because it gives rise to higher amounts of RISC bound sCAGs.

## Discussion

Our data confirm previous results that the regions that contain extended (CAG)_*n*_ in both *HTT* and *AR* and form HPs are stabilized by adjacent nonCAG TNR sequences that act as clamps [35, 68]. In addition, they suggest that both the *HTT* and the *AR*-derived HPs are toxic to cells through RNAi. Both HPs depend on Dicer for processing and AGO2 to mediate RNAi. Our data also suggest that the LOI mutant HTT is more toxic than the Ref sequence and this is based on its unique structure with much longer CAG TNR sequences that are part of an extended double stranded stem region without an interruption by a loop region. This may make this structure a better substrate for Dicer resulting in an uptake of a larger number of CAG containing short RNAs into the RISC. Longer double stranded (CAG)_*n*_ extensions in *HTT* will therefore result in higher amounts of RISC bound sCAG and hence higher toxicity.

Recently, the data on the role of the length of uninterrupted CAG mRNA rather than the length of the polyQ stretch was confirmed in a new transgenic mouse model [70]. These bacterial artificial chromosome (BAC) transgenic mice express human mutant huntingtin (m*HTT*) with uninterrupted CAG repeats (BAC-CAG mice). By comparing these mice with multiple other HD mouse models carrying CAA-interrupted CAG repeats a robust positive correlation between the average concordance and uninterrupted mutant huntingtin CAG repeat length was found, whereas the correlation with glutamine repeat length was not statistically significant. Interestingly, while it was mentioned that CAG containing short RNAs can be toxic to cells, the toxicity of the CAG repeat containing RNAs was mostly discussed in the context of RAN translation and of their association with nuclear foci formation and colocalization with MBNL1 rather than through the RNAi activity of small CAG repeat containing RNAs.

MBNL1 binds to double stranded CUG repeat regions [72]. It is believed that via this activity MBNL1 contributes to the formation of nuclear CUG RNA foci, and that nuclear but not cytoplasmic localization triggers pathogenesis in the CUG repeat disease Myotonic dystrophy type 1 (DM1) [73]. There is, however, evidence showing that such foci do not contribute to disease pathology [74]. Furthermore, experimental results show that structures formed by CAG TNRs are susceptible to RNAi, suggesting that these HPs are transported to the cytosol where most of the RISC complexes are located [35, 68] and where they can become RNAi active. Our data suggest that HPs mimicking the RNA structures that form in m*HTT* or m*AR* are toxic to cells through RNAi. Based on our finding that a HP resembling the HTT LOI mutant is more toxic and produces more sCAG than the Ref mHTT, we provide an alternative explanation for how only two point mutations in m*HTT* in the LOI variant can result in a 25 year earlier age at onset of disease. Our results support the idea that targeting sCAGs rather than the entire mCAG-RNA would be a good approach to treating these diseases, as this would selectively reduce the amount of disease-causing sCAGs without affecting the mRNA levels of the wild-type HTT mRNA. An allele specific targeting would therefore not be necessary when inhibiting sCAGs in diseases caused by CAG repeat extensions.

## Supplementary information

Supplemental material

Reproducibility checklist

## Data Availability

The data that support the findings of this study are available from the corresponding author upon reasonable request.
