# Supplementary material for: The length of uninterrupted CAG repeats in stem regions of repeat disease associated hairpins determines the amount of short CAG oligonucleotides that are toxic to cells through RNA interference
Source: Cell Death Dis. 2022 Dec 30;13(12):1078. doi: 10.1038/s41419-022-05494-1 (PMC9803637; doi:10.1038/s41419-022-05494-1)
Supplement: Supplementary file 1 — Supplemental material [file 41419_2022_5494_MOESM1_ESM.pdf]

A **293T**

Gene name	Length of (CUG)n in nts	Signal
HNRNPU*	11	1299
RPLP0*	13	1233
RPL13*	13	1161
HSPA8**	11	1008
NUCKS1	11	1006
RPLP1*	11	883
AMOT	≥19	843
GNAS**	12	804
RPL14	≥19	745
NAP1L1	11	717

B **HeLa**

Gene name	Length of (CUG)n in nts	Signal
RPLP0*	13	2260
ALPI	13	1984
RPL13*	10	1213
RPLP1*	11	1207
HNRNPU*	11	1149
HSPA8**	11	1117
AHNAK	11	1065
GNAS**	12	1013
HSPA5	13	967
CD55	13	944

C **Human brain**

Gene name	Length of (CUG)n in nts	Signal
GFAP	≥19	3726
KIF5A	11	1791
GNAS**	12	1562
HSPA8**	11	832
RTN1	11	830
UCHL1	13	694
APLP1	11	631
NCS1	10	615
NTRK2	13	532
PKD1	10	453

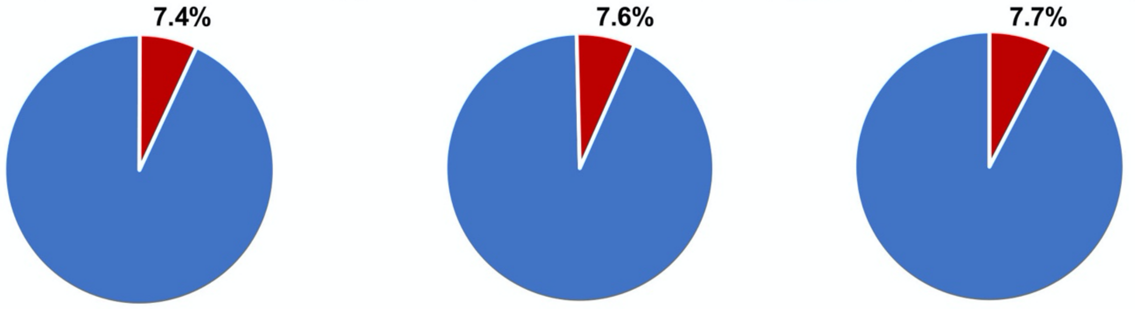

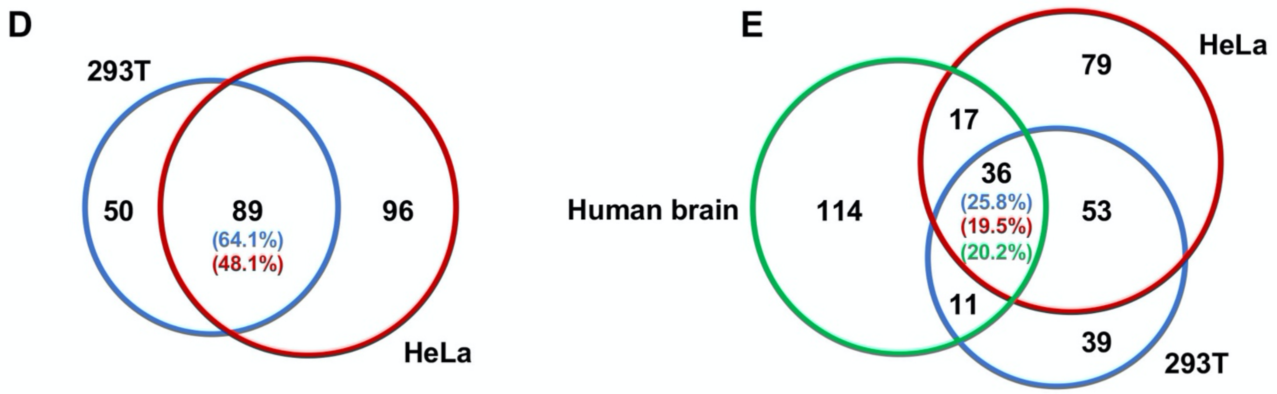

Supplementary Figure 1. Highly expressed CUG repeat containing genes in human brain, HeLa and 293T cells. **A**, **B***,* **C** *top panels,* ranked lists of the most highly expressed genes in 293T cells (A), HeLa cells (B), and human brains (C) containing CUG repeats in their mRNA. The length of the longest $(CUG)_n$ present is given for each gene in nucleotides as well as the expression levels of coding genes (normalized to 1 million). DepMap survival genes are shown in red. *Genes found in more than one analysis; **genes found in all three analyses. **A**, **B***,* **C** *bottom panels,* contribution of expressed mRNAs (in percent) with CUG repeats to the total expressed genes in each of the three data sets. **D** Venn diagram showing the overlap of the $(CUG)_n$ containing genes in the two cell lines. **E** Venn diagram showing the overlap of the $(CUG)_n$ containing genes in the two cell lines and human brain. The percent overlap refers to the data set in the same color. From each data set the most highly expressed genes (normalized signal >100) was used. Data were extracted from GSE171397 and GSE209928, and GSE64810. Data from unmanipulated cells or normal brains were used.

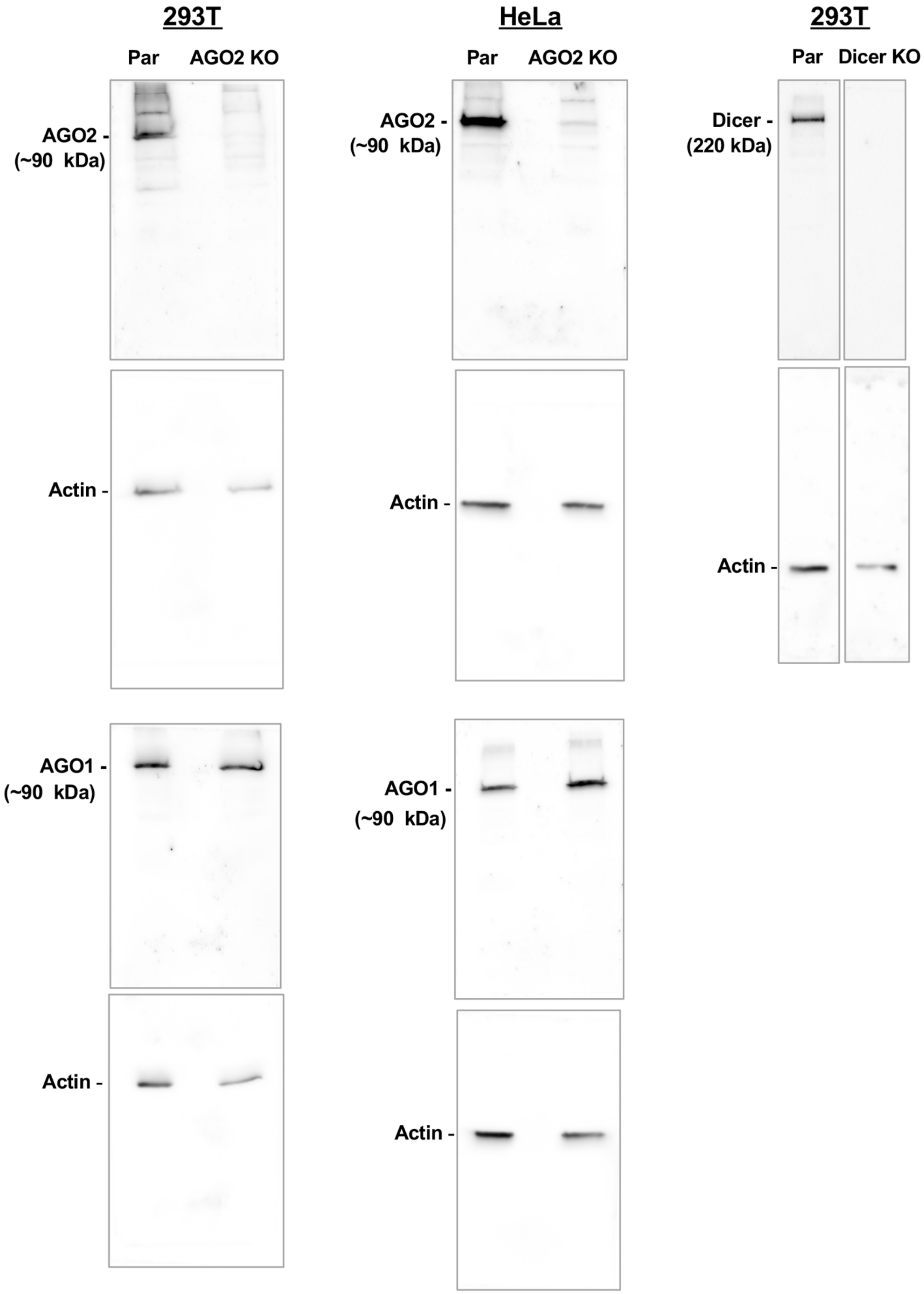

Supplementary Figure 2. Uncropped Western blots of Figure 3.
